# Supplementary material for: Combined Naltrexone–Bupropion Therapy for Concurrent Cocaine Use Disorder and Obesity: A Case Report
Source: Reports (MDPI). 2025 Sep 8;8(3):174. doi: 10.3390/reports8030174 (PMC12452683; doi:10.3390/reports8030174)
Supplement: Supplementary file 1 [file reports-08-00174-s001.zip › Table_S1_CARE_Checklist.pdf]

Table S1. CARE Checklist for Case Report Compliance

Legend: Y = present in manuscript; N = not included; NA = not applicable. Section references indicate where each item appears in the manuscript.

CARE Checklist Item	Recommendation (abridged)	Where in Manuscript	Status	Notes
**Title**	The words 'case report' appear in the title; key phenomenon included.	Title page	Y	Compliant with updated title.
**Keywords**	3–10 pertinent keywords.	Keywords	Y	Alphabetical, semicolon-separated.
**Abstract**	Structured abstract with Background/Significance, Case Presentation, Conclusions.	Abstract	Y	≈250 words, structured.
**Introduction**	Brief background and rationale.	Introduction	Y	Focused rationale for NB.
**Patient information**	De-identified demographics; main concerns; relevant medical/family/psychosocial history; prior interventions.	Case Presentation	Y	Identifiers removed.
**Clinical findings**	Pertinent physical exam and clinical data.	Case Presentation	Y	BMI, labs, ECG reported.
**Timeline**	Chronological timeline of care.	Case Presentation (Table)	Y	Weeks 0–12 dosing and outcomes table.
**Diagnostic assessment**	Methods; diagnostic reasoning; prognostic characteristics; challenges.	Case Presentation	Y	DSM-5 CUD; toxicology.
**Therapeutic intervention**	Type; dosage; administration; changes.	Therapeutic intervention and time-line	Y	NB titration to 32/360 mg; CBT; lifestyle.
**Follow-up and outcomes**	Adherence; tolerability; clinical outcomes; adverse events.	Outcomes	Y	Abstinence, scales, BMI, UKU.
**Discussion**	Strengths/limitations; literature context; rationale for conclusions.	Discussion	Y	Updated ≥2020 literature.

**Patient perspective**	Patient's experience/perspective.	—	N	Optional but recommended; not collected.
**Informed consent**	Statement confirming written informed consent to publish.	Ethical Declarations and Consent	Y	Included verbatim.
**Ethics approval**	IRB approval or waiver with justification.	Ethical Declarations and Consent	Y	Waived; non-interventional de-identified case.
**Confidentiality/Privacy**	Measures to protect identity; media/images.	Ethical Declarations and Consent	Y	No identifying details/images.
**Funding**	Sources of support, if any.	Back Matter	Y	No external funding.
**Author Contributions (CRediT)**	Contributor roles listed.	Back Matter	Y	Single author (V.M.R.).
**Conflicts of Interest**	Disclosure of conflicts.	Back Matter	Y	None declared.
**Data availability**	Statement on data sharing.	Back Matter	Y	De-identified data on request.
**CARE checklist**	Completed CARE checklist provided as Supplementary Material.	Supplementary Material (this table)	Y	Submitted as Table S1.

This checklist follows the CARE Guidelines (Riley et al., 2017) and MDPI Reports author guidance.
