# Supplementary figures and images for: Combined Naltrexone–Bupropion Therapy for Concurrent Cocaine Use Disorder and Obesity: A Case Report
Source: Reports (MDPI). 2025 Sep 8;8(3):174. doi: 10.3390/reports8030174 (PMC12452683; doi:10.3390/reports8030174)

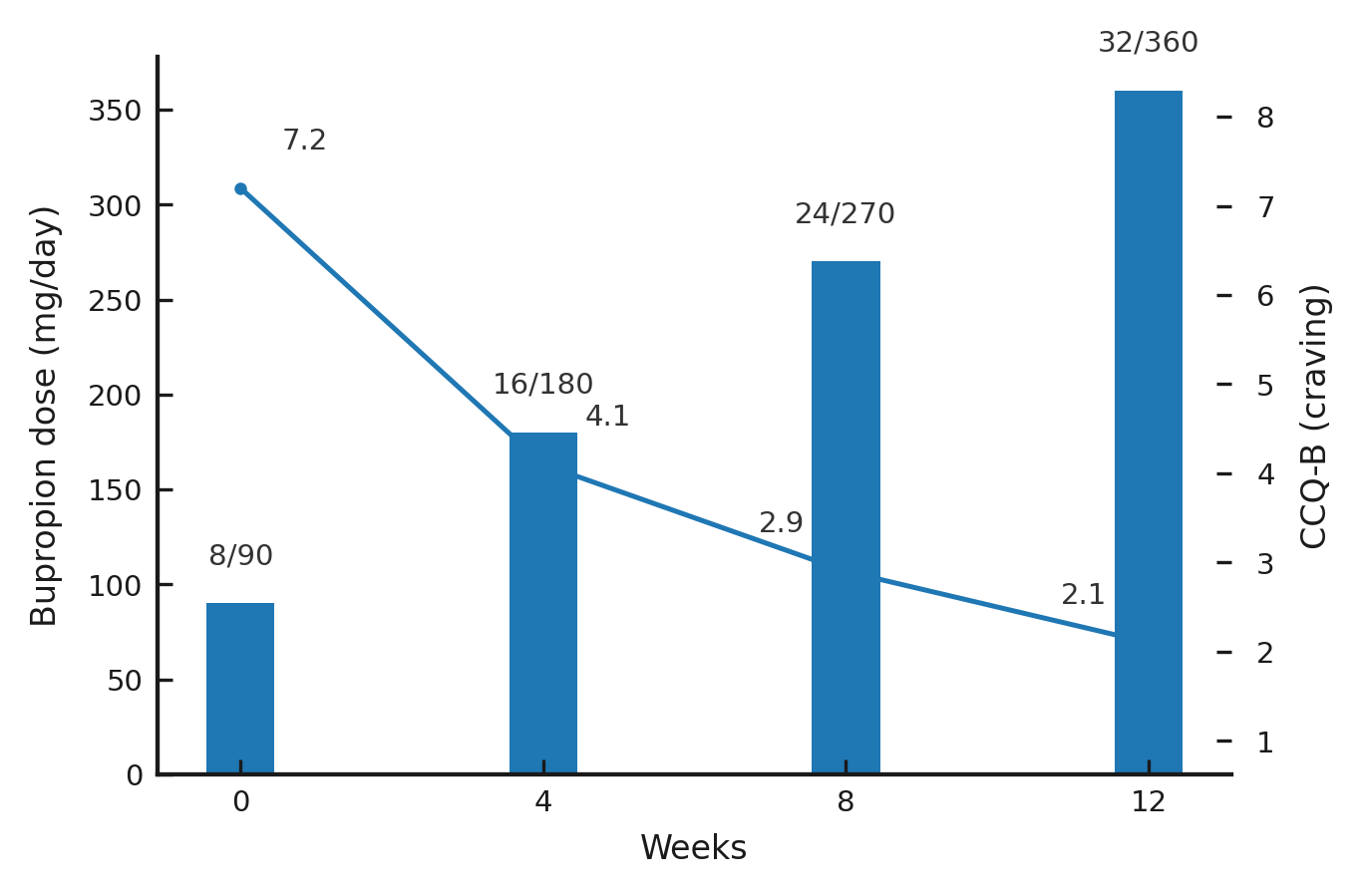

Supplement: Supplementary file 1 [file reports-08-00174-s001.zip › Figure_S1_Dose_CCQB_Overlay_v2 copia.png]
